# Electroacupuncture Produces the Sustained Motor Improvement in 6-Hydroxydopamine-Lesioned Mice

**DOI:** 10.1371/journal.pone.0149111

**Published:** 2016-02-19

**Authors:** Yan Yu, Ke Wang, Jiahui Deng, Min Sun, Jun Jia, Xiaomin Wang

**Affiliations:** Departments of Neurobiology and Physiology, Key Laboratory for Neurodegenerative Disorders of the Ministry of Education, Beijing Key Laboratory for Parkinson’s Disease, Capital Medical University, Beijing Institute for Brain Disorders, Beijing, 100069, China; National Institute of Health, UNITED STATES

## Abstract

Clinical and research evidence has shown that electroacupuncture (EA) promotes recovery of motor function in patients with Parkinson’s disease (PD). However, the “efficacy span” of EA treatment, especially the long-term effect of EA that is thought to last after the cessation of EA treatment, has not been investigated. The present study thus investigated and compared the effect of EA during and after chronic EA application on motor activity and dopamine lesions in a 6-hydroxydopamine (6-OHDA)-lesioned mouse model of PD. Chronic EA treatment (30 min a day, 6 days a week for 2 or 4 weeks) significantly attenuated motor deficiency and reduced dopamine neuron degeneration. Remarkably, EA showed a long-lasting effect after the cessation of EA stimulation. At 2 and 4 weeks after the termination of EA, EA continued to improve motor function in 6-OHDA-lesioned mice. Consistent with sustained behavioral effects, EA induced an enduring increase in the dopamine turnover ratio in the striatum 2 weeks after the cessation of EA treatment. Here we demonstrated that the therapeutic effect of EA outlasted the duration of EA application. During a relatively long period of time after the completion of EA treatment, EA is able to continue to improve motor function and enhance dopamine availability in 6-OHDA-lesioned PD mice.

## Introduction

Parkinson’s disease (PD) is one of the most common neurodegenerative diseases in the elderly. It mainly affects the motor system and at a late stage it also causes other psychiatric symptoms. The loss of dopamine (DA) neurons projecting from the substantia nigra (SN) to the striatum is believed to be responsible for many motor deficits of PD, including resting tremor, rigidity, slowness of movement and difficulty with walking and gait. In the absence of perfect strategy in treating PD, all the efforts were made to alleviate the symptoms or improve the life quality of PD patients [1]. Although classical medical administration of levodopa (L-Dopa) and surgical therapeutic options, such as deep brain stimulation (DBS), are playing the most important role in alleviating the motor syndrome, PD patients have to face-up to the inevitable side effects and financial burden [2,3]. Nowadays, Acupuncture or electroacupuncture (EA, also known as peripheral electrical stimulation) represents a non-invasive complementary therapy and has long been used to alleviate the symptoms of PD patients [4,5].

Accumulating evidence has suggested that EA treatment attenuates PD-related symptoms in human and animal subjects [5–7]. It has been found that acupuncture treatment significantly increased the total Unified Parkinson Disease Rating Scale score of PD patients [8]. Our previous studies have also demonstrated that high-frequency EA (100 Hz) stimulation significantly reduced abnormal behaviors and enhanced recovery of the dopaminergic nigrostriatal system in rodent’s PD models, including medial forebrain bundle (MFB) axotomy in rats [9], 6-hydroxydopamine (6-OHDA)-induced lesion of MFBs in rats [10], and MPTP-induced dopamine depletion in mice [11].

While the therapeutic effect of EA has been well demonstrated, all early studies have been focused on the effect of EA that was produced during the period of EA stimulation or shortly after EA stimulation. This leaves an important question unanswered, i.e., whether the EA effect can sustain after the cessation of EA stimulation. Since the answer to this question determines the temporal nature of EA action and the usefulness of EA in treating PD patients [12,13], It is worthwhile to investigate and characterize the effect of EA after EA stimulation is terminated.

In this study, we thus set forth to investigate the long-lasting effect of EA. We used a mouse model of PD in which the unilateral lesion of the nigrostriatal DA system was made by injection of 6-OHDA toxin, even though this is an imperfect model regarding the loss of DA. The effect of EA on motor symptoms and dopaminergic degeneration was examined after termination of EA application. DA contents and metabolites were measured to explore the possible mechanism underlying the sustained effect of EA.

## Materials and Methods

### Animals

Male C57BL/6J mice weighting 20–25 g (8 weeks old) were provided by the animal facility of Capital Medical University. Mice have free access to food and water *ad libitum* with a 12 h/12 h light/dark cycle. All experimental procedures followed the principles of the Chinese Specifications for the Production, Care and Use of the Laboratory Animals. The animal use and care protocol was approved by Committee on Animal Care and Usage of Capital Medical University.

### Experimental design

**Fig 1:**
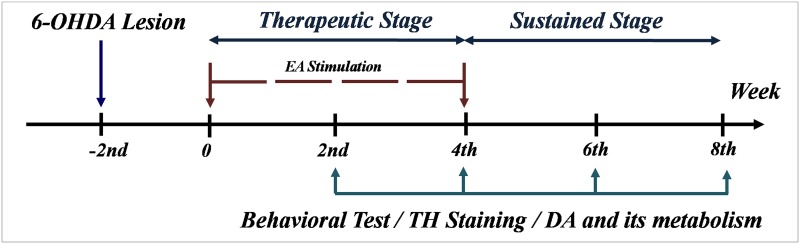
Graphical representation of the experimental design. After 2 weeks post-lesion by 6-OHDA, the mice were subjected to the EA stimulation (30 min a day, 6 days a week for a total of 4 weeks). Behavioral test, immunohistochemistrical staining for tyrosine hydroxylase (TH), content of DA and its metabolites were performed at the end of 2^nd^, 4^th^, 6^th^ and 8^th^ week.

A schematic diagram of experiments is present in Fig 1. Generally, mice were randomly divided into 4 groups: a sham group, a 6-OHDA-lesioned group, and two groups of 6-OHDA-lesioned mice receiving EA at either 0 or 100 Hz. Two weeks after 6-OHDA injection, EA stimulation was administrated to unilateral 6-OHDA-lesioned mice (30 min a day, 6 days a week for a total of 4 weeks). The effect of EA on motor activities, including rotarod, cylinder and locomotor activity, was tested at four different time points: 2 or 4 weeks after the start of EA stimulation referred to a ‘therapeutic stage’ and 2 or 4 weeks after the cessation of EA stimulation, i.e., 6 or 8 weeks after the start of EA stimulation, referred to a ‘sustained stage’. At four time points, mice were sacrificed to determine the tyrosine hydroxylase (TH) staining and analyze changed in dopamine (DA) and its metabolites. The behavior tests including rotarod, cylinder and locomotor activity were performed every two weeks by assessors of randomization and blinding.

### Unilateral 6-OHDA lesion

Mice were anaesthetized by an intraperitoneal (i.p.) injection of 6% chloral hydrate (350 mg/kg) and placed into a Kopf stereotaxic apparatus. 6-hydroxydopamine hydrobromide (Sigma-Alorich) was injected in the two deposits in the striatum at the following coordinates (relative to bregma): AP, 0.5 mm; ML, -2.0 mm, DV: -3.0 mm and then DV: -2.0 mm from the dura. At each site, 6-OHDA (8 μg) was injected at in a total volume of 0.8 μl (5 μg/μl) at a rate of 0.5 μl/min with a 10 μl microsyringe. The microsyringe was left for additional 3 min after the injection. Animals were allowed 2 weeks for recovery before EA experiments.

A total of 250 mice were used in experiments with unilateral 6-OHDA striatal lesions. There was approximately a 70% success rate with a mortality rate of 15% in this study. Sham-operated control mice underwent the same surgical procedures except that saline was injected. Animal health and behavior were monitored daily following the 6-OHDA lesions. All mice were subjected to behavioral analysis every two weeks in all tests described below and showed a steady increase in their body weight and food intake following the 6-OHDA lesions.

### EA stimulation

EA stimulation as we described previously [11,14]. In brief, two stainless steel needles (0.18 mm in diameter and 3 mm in length) were inserted across the acupoint Zusanli (ST36, 2 mm lateral to the anterior tubercle of tibia) and Sanyinjiao (SP6, 2 mm proximal to the upper border of medial malleolus, at the posterior border of the tibia) of each hindlimb[15]. Bidirectional square wave electrical pulses (0.2 ms duration, 100 Hz) generated from a Han's acupoint nerve stimulator (HANS, manufactured Neuroscience Research Institute, Peking University) were connected to the stainless steel needles in both hindlimbs simultaneously. Mice were gently handled and lightly restrained in a plastic cylinder (7 × 2.5 cm) with their hind limbs accessible for needling. For this procedure, the mice were immobilized for 30 minutes during EA administration with no anesthesia [14]. The intensity of the stimulation was stepwise increased from 1.0 mA to 1.2 mA and then to 1.4 mA. Each step lasted for 10 min. The mice remained relaxed and didn’t show stress-like behaviors such as vocalization and hindlimbs flinches during the whole electrical stimulation. EA stimulation was given for a total of 30 min each day, 6 days per week. The duration of EA treatment was limited to 4 weeks. The mice treated with EA at 0 Hz underwent the same procedures, but no electrical pulses were delivered through the needles. Consistent with our previous studies [11,16], EA at 0 Hz was used as a procedure control. Our previous study has been showed EA stimulation at non-acupoins has not any effect of motor improvement [10]. Therefore, in this study we didn’t show any data of non-acupoints more.

### Rotarod

A rotarod apparatus (Stoelting Company, wood Dale, IL) was used to assess the balance and motor coordination of mice [17]. Before the 6-OHDA lesion surgery, all mice were trained on the rotarod in order to reach a stable performance. The training was performed on three consecutive days. On the test day, accelerating rotarod (maximum speed at 40 rotations per minute (rpm) was performed. The time taken to fall was automatically recorded upon landing of fallen mice on the base of the apparatus.

### Cylinder Test

The cylinder test was used to assess the forelimb asymmetry in a novel environment [18]. Mice were placed in a glass beaker (height: 14 cm, diameter: 5 cm) and videotaped for 5 min without habituation. The number of independent, weight-beating contacts on the cylinder wall of ipsilateral paw, contralateral paw or simultaneous paws, relative to the lesioned hemisphere was scored. Data were showed as a percentage of contralateral touches, and calculated by the contralateral touches / (contralateral touches + ipsilateral touches + simultaneous touches).

### Locomotor activity

Horizontal and vertical locomotor activities were assessed by an automated system (Tru Scan system, Coulbourn Instruments, USA) as previously described [11]. Mice were placed in a closed individual cage (25.4 × 25.4 inch) which contains a grid of infrared beams mounted horizontally every 2.5 cm. Spontaneous locomotor activity was recorded as the total movement distance (cm). All experiments were performed from 9:00 to 12:00 am and lasted for 30 min. The environment was kept dark and quiet during entire procedures.

### Immunohistochemistry

Mice were anesthetized and transcardially perfused with 0.9% sodium saline (room temperature), followed by 4% paraformaldehyde/phosphate-buffered saline (PBS) (pH 7.4, ice-cold). The brain was dissected and post-fixed in the 4% paraformaldehyde/PBS overnight. The brain was transferred to 20% and 30% sucrose solution separately for tissue cytoprotection. Coronal sections (30 μm) were cut at by a freezing microtome (Leica) and stored in an antifreeze solution at 4°C. Sections of the substantia nigra or striatum were collected for immunohistochemistry which was performed as described previously [11]. In brief, sections were incubated in a mouse anti-TH antibody (1:2000, Sigma, USA) overnight at 4°C. After rinsing three times with PBS, sections were respectively incubated with a second antibody (1:200) and AB work solution (1:1:100) for 30 min at 37°C (Vector Laboratories, USA). DAB solutions (a drop DAB to 2 ml substrate liquid) was used to visualize the antibody. Sections were fixed on slides and covers lipped with a water-soluble mounting medium.

### Quantification of TH immunoreactive cell in substantia nigra pars compacta (SNpc) and TH immunoreactive fibers in the striatum

The number of TH immunoreactive cell bodies in the SNpc was counted by stereology counting with the stereo investigator software (MBF bioscience, USA) [19]. The average number of SNpc sections was six in a 1:6 series. The counting of the number of TH cell bodies was calculated using a 20 × objective lens on a Leica microscope. Quantification of TH cell bodies was expressed as a percentage of numbers in the lesioned side relative to that in the unlesioned side. Images of TH-stained striatal sections were obtained by an Olympus microscope. Immunoreactive optical densities of TH fibers in the striatum were calculated using the Image-Pro Plus software 6.0. Quantification of TH fibers was expressed as a percentage of density in the lesioned side relative to that in the unlesioned side.

### High performance liquid chromatography (HPLC) analysis of dopamine and its metabolites

Content of DA and its metabolites, 3,4, dihydroxyphenylacetic acid (DOPAC) and homovanillic acid (HVA), in the striatum were detected by HPLC-ECD (Model 5600A Coul Array Detector System, ESA Inc., USA) [10]. The striatum was rapidly dissected, frozen in liquid nitrogen, and stored at -80°C. Striatal tissue was weighed and homogenized in 200 μl perchloric acid (0.4 M). Homogenates were centrifuged for 20 min at 12,000 rpm at 4°C after ice-bath for 60 min. Supernatant (160 μl) was mixed with 80 μl mobile phase solution and was kept from light for ice-bath (60 min), followed by centrifugation (12,000 rpm, 20 min). The resultant supernatant was filtered with a 0.22 mm membrane. An aliquot (20 ml) of the resulting solution was injected into the HPLC pump with four potentials of -150, 100, 220 and 400 mV and flow rate at 1 ml/min. Chromatographic separation was performed using a HR-C18 reverse-phase column (80 × 4.6 mm I.D., 3 μm, 100A, ESA Inc., USA). Data analysis was performed using analysis software (CoulArray for Wingdows^®^32 application software, ESA Inc., USA). The mobile phase (pH4.3) contains 63.5 mM citric acid monohydrate, 60.9 mM trisodium citrate dihydrate, 0.1 mM EDTA, 0.5 mM 1-octanesulfonic acid sodium salt, and 8% methanol. DA, DOPAC, and HVA were prepared in 0.1% perchloric acid. Each concentration was adjusted with respect to the standard and quantified from a standard curve. Levels of DA, DOPAC, and HVA were calculated as micrograms per milligram of total wet tissue.

### Statistical analysis

Statistical analysis was performed by GraphPad Prism 5.0. All values in the text and figures are presented as mean ± SEM. All data were subjected to ANOVA followed by the New-man Keul's post hoc. Probabilities of 0.05 or less were considered statistically significant.

## Result

### Effects of EA on motor function and dopaminergic neurodegeneration at the therapeutic stage

**Fig 2:**
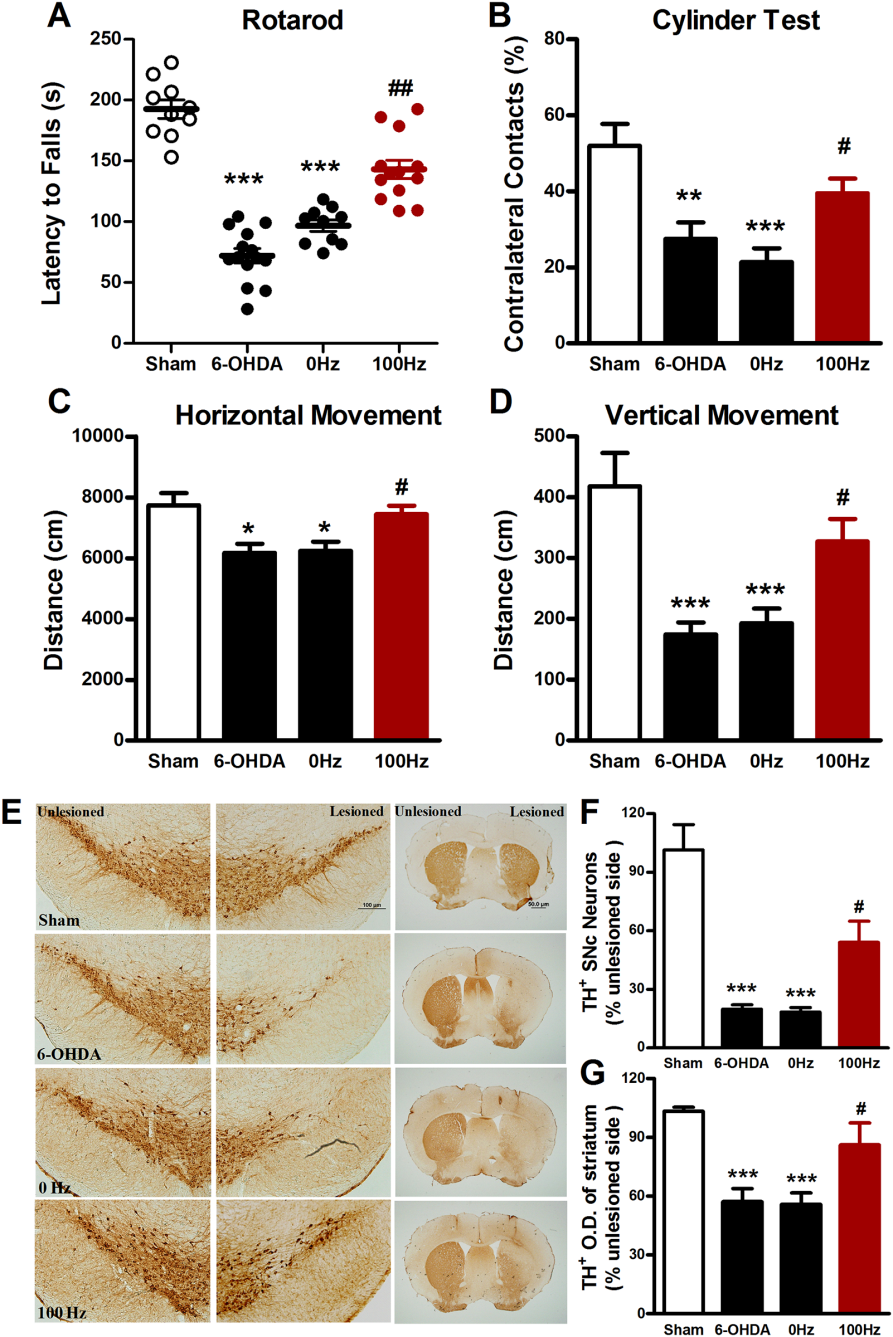
Two weeks’ EA stimulation exerted the behavioral improvements and neuroprotective effects on 6-OHDA-lesioned mice. (A) Rotarod test. The time to fall off the rotarod treadmill was recorded as the latency. (B) Cylinder test. The percentage of contralateral paw contacts relative to the lesioned hemisphere was calculated by contralateral touches / (contralateral touches + ipsilateral touches + simultaneous touches). Locomotor activity was assessed by horizontal (C) and vertical (D) movement distance. (E) Immunohistochemistrical staining for TH positive dopaminergic neurons in the SNpc (scale bar: 100 μm) and fibers in the striatum (scale bar: 50 μm). (F) The percentage of TH positive dopaminergic neurons from the SNpc in the lesioned side relative to that in the unlesioned side. (G) Optical density was used to evaluate relative TH expression from lesioned striatum compared with unlesioned one. The values were expressed as means ± SEM. n = 10–12 per group for the behavior tests; n = 5 per group for the immunohistochemical experiments. * *P* <0.05; ** *P* <0.01; *** *P* <0.001 *vs*. Sham group. # *P* <0.05; ## *P* <0.01 *vs*. 0 Hz group.

**Fig 3:**
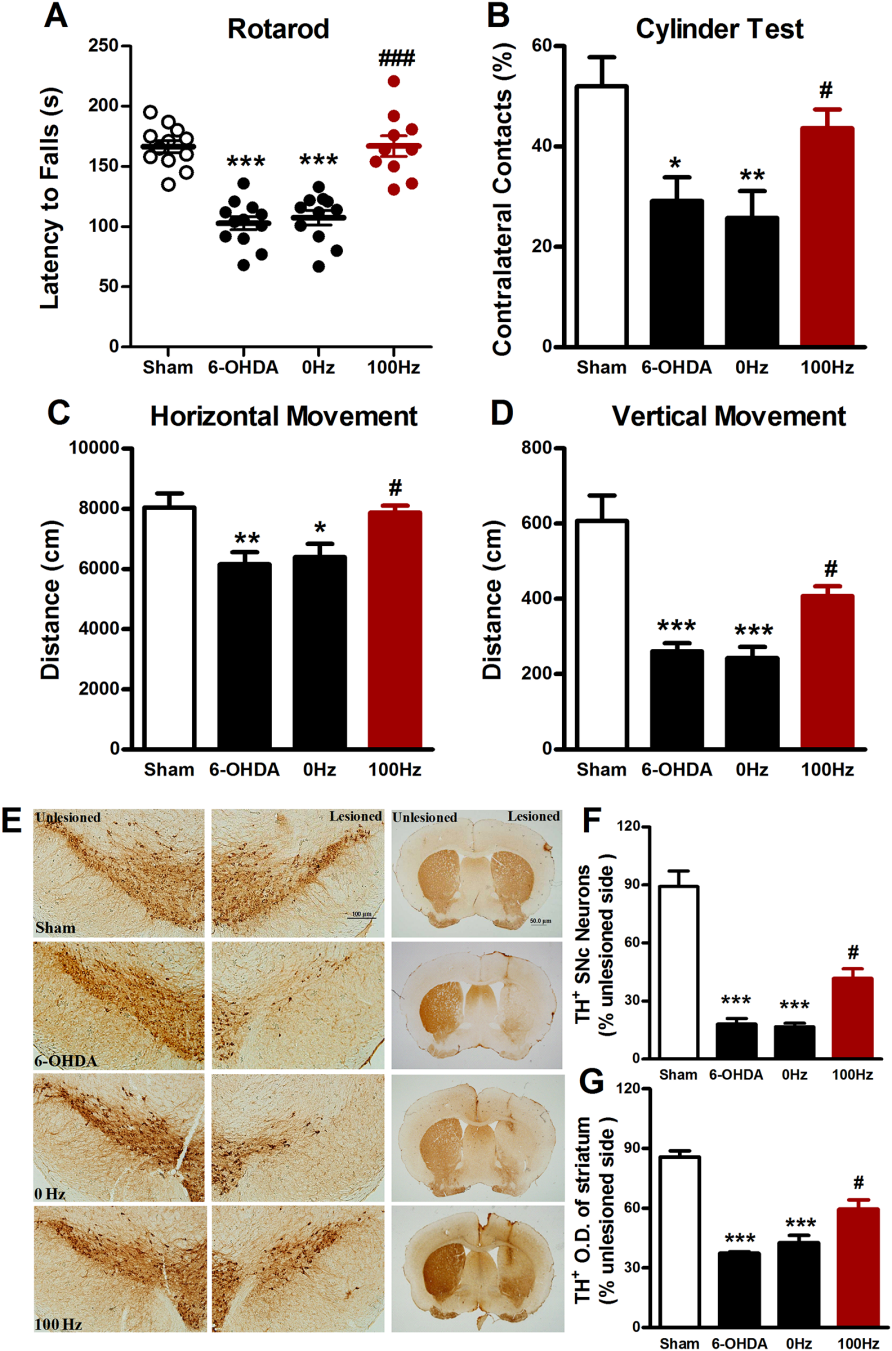
Four weeks’ EA stimulation exerted the behavioral improvement and neuroprotective effects on 6-OHDA-lesioned mice. (A) Rotarod test. (B) Cylinder test. (C) Horizontal locomotor distance. (D) Vertical locomotor distance. (F) Immunohistochemistrical staining for TH positive dopaminergic neurons in the SNpc (scale bar: 100 μm) and fibers in the striatum (scale bar: 50 μm). (G) The percentage of TH positive dopaminergic neurons in the SNpc (H) The percentage of average optical density of TH positive dopaminergic fibers in the striatum. The data were expressed as means ± S.E.M. n = 10–12 per group for the behavior tests; n = 5 per group for the immunohistochemistrical experiments. The values were expressed as means ± SEM. * *P* <0.05; ** *P* < 0.01; *** *P* <0.001 *vs*. Sham group. # *P* <0.05; ### *P* <0.001 *vs*. 0 Hz group.

**Fig 4:**
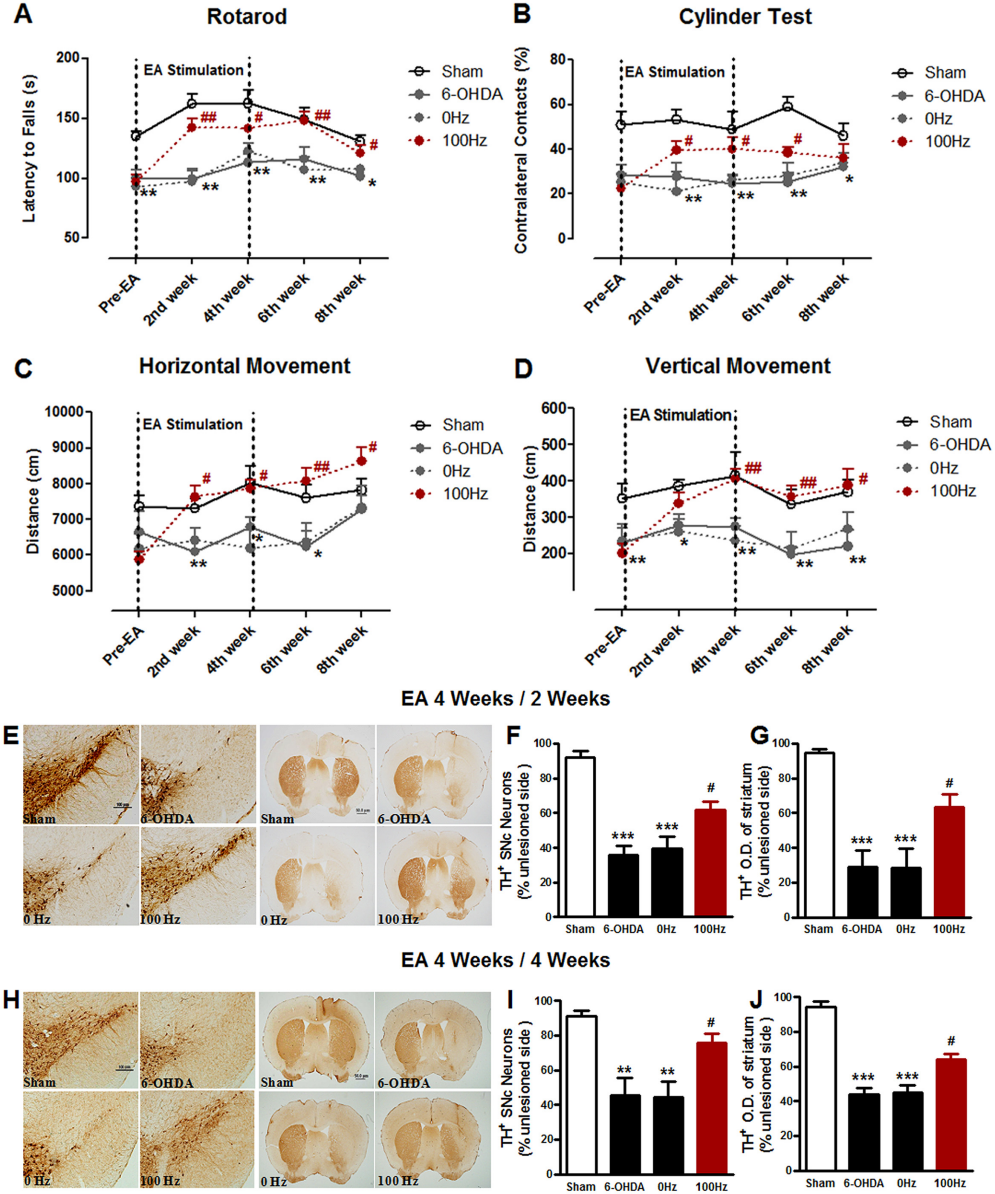
EA produces a long-last neuroprotection in 6-OHDA-lesioned mice after the termination of EA stimulation. Time-dependent alteration of the behavioral performance in each group, such as rotarod test (A), cylinder test (B), horizontal locomotor distance (C) and vertical locomotor distance (D). (E) immunohistochemistrical staining for TH positive dopaminergic neurons in the SNpc (scale bar: 100 μm) and fibers in the striatum (scale bar: 50 μm) were showed at 2^nd^ and 4^th^ week after the termination of EA stimulation (which be described as EA 4 Weeks / 2 Weeks and EA 4 Weeks / 4 Weeks, respectively). Bar graph of the percentage of TH positive dopaminergic neurons in the SNpc (F) and the fibers in the striatum (G) at the end of 2^nd^ week after the termination of EA stimulation. At the 4^th^ week after the termination of EA stimulation, the percentage of TH positive dopaminergic neurons in the SNpc was shown as the bar graph (H), and the fibers in the striatum was shown as the bar graph (I). n = 10–12 per group for the behavior tests; n = 5 per group for the immunohistochemistrical experiments. The values were expressed as means ± SEM. * *P* <0.05; ** *P* <0.01; *** *P* <0.001 *vs*. Sham. # *P* <0.05; ## *P* <0.01 *vs*. 0 Hz group.

As illustrated in Figs 2 and 3, in 6-OHDA-lesioned mice, effects of EA on motor activities and TH immunostaining were investigated at the therapeutic stage, i.e., 2 and 4 weeks after the start of EA stimulation. We found that 100 Hz EA significantly increased the latency in the rotarod test at both 2 (Fig 2A) and 4 (Fig 3A) weeks, while 0 Hz EA did not, but 100 Hz EA also increased the percentage of contralateral forelimb placement at 2 weeks (39.5 ± 3.8% *vs*. 21.3 ± 3.7%, *P* < 0.05, Fig 2B) and 4 (43.7 ± 3.7% *vs*. 25.8 ± 5.4%, *P* < 0.05, Fig 3B) weeks after the start of EA stimulation. Similar results were observed in the locomotor behavioral test. A significant increase in the total movement distance in both horizontal and vertical directions was seen in mice treated with 100 Hz EA as compared to mice treated with 0 Hz EA at both 2 (Fig 2C and 2D) and 4 (Fig 3C and 3D) weeks.

Immunohistochemistrical data showed that intra-striatal injection of 6-OHDA induced a loss of TH-positive cells by 80.1 ± 2.2% in the SNpc in the injected side. 100 Hz EA stimulation for two weeks rescued the loss of TH-positive cells to 54.0 ± 10.9% of sham control (Fig 2E and 2F). Densitometry analysis revealed that two weeks of 100 Hz EA reversed the reduction of TH-positive fiber density (Fig 3E and 3G). Similarly, TH-staining assay showed that 4 weeks of 100 Hz EA increased the number of TH positive cells (41.5 ± 4.9% of 100 Hz group *vs*. 16.8 ± 1.8% of 0 Hz group, *P* < 0.05, Fig 3E and 3F) and restored TH positive fiber density (59.5 ± 4.7% of 100 Hz group *vs*. 39.5 ± 3.6% of 0 Hz group, *P* < 0.05, Fig 3E and 3G). As one of phenotypic markers of the nigrostriatal dopaminergic system, the loss of TH reflects a change in dopaminergic phenotype [20]. The data obtained here suggest that 100 Hz EA stimulation at two time points defined for a therapeutic stage, i.e., 2 and 4 weeks after the start of chronic EA application, can reduce the loss of TH in 6-OHDA-lesioned mice.

### Effects of EA on motor function and dopaminergic neurodegeneration at the sustained stage

As a major aim of this investigation, the effect of EA was tested 2 and 4 weeks after the cessation of four-week EA stimulation. As shown in Fig 4A–4D, the EA-induced motor improvement persisted at 2 weeks after the termination of EA. A significant increase in rotarod motor coordination (Fig 4A) and spontaneous locomotion (Fig 4C and 4D) and a decrease in forelimb akinesia in the cylinder test (Fig 4B) were observed at the early sustained stage (2 weeks) in mice treated with 100 Hz EA relative to mice treated with 0 Hz EA. At 4 weeks after the termination of EA stimulation, motor improvements as measured by rotarod (Fig 4A) and locomotion (horizontal movement, Fig 4C; vertical movement, Fig 4D) were still seen in mice treated with 100 Hz EA. Only in the Cylinder test, 100 Hz EA showed no significant effect at 4 weeks after the completion of EA stimulation. Thus, EA generally produces a long-last motor improvement in 6-OHDA-lesioned mice at the sustained stage.

Immunostaining for TH was also performed 2 and 4 week after the termination of EA stimulation. In mice treated with 0 Hz EA, the numbers of TH positive neurons in the lesioned side were approximately 35.6 ± 5.7% of that in the unlesioned side. The number of TH positive neurons in mice treated with 100 Hz EA was significantly increased to 61.9 ± 4.7% at 2 weeks (Fig 4E and 4F) or 77.4 ± 5.1% at 4 weeks (Fig 4H and 4I) after the termination of EA stimulation. In the striatum, 100 Hz EA also showed the similar protection of the dopaminergic fibers against 6-OHDA at either 2 weeks (63.5 ± 7.4% of 100 Hz group *vs*. 28.6 ± 10.9% of 0 Hz group, *P* < 0.05 Fig 4E and 4G) or 4 weeks (64.1 ± 3.2% of 100 Hz group *vs*. 45.1 ± 4.2% of 0 Hz group, *P* < 0.05, Fig 4H and 4J) after the termination of EA stimulation. These results indicate that the improved effects of EA on dopaminergic phenotype can sustain up to four weeks post EA stimulation.

### The sustained effect of EA on DA turnover ratio

**Fig 5:**
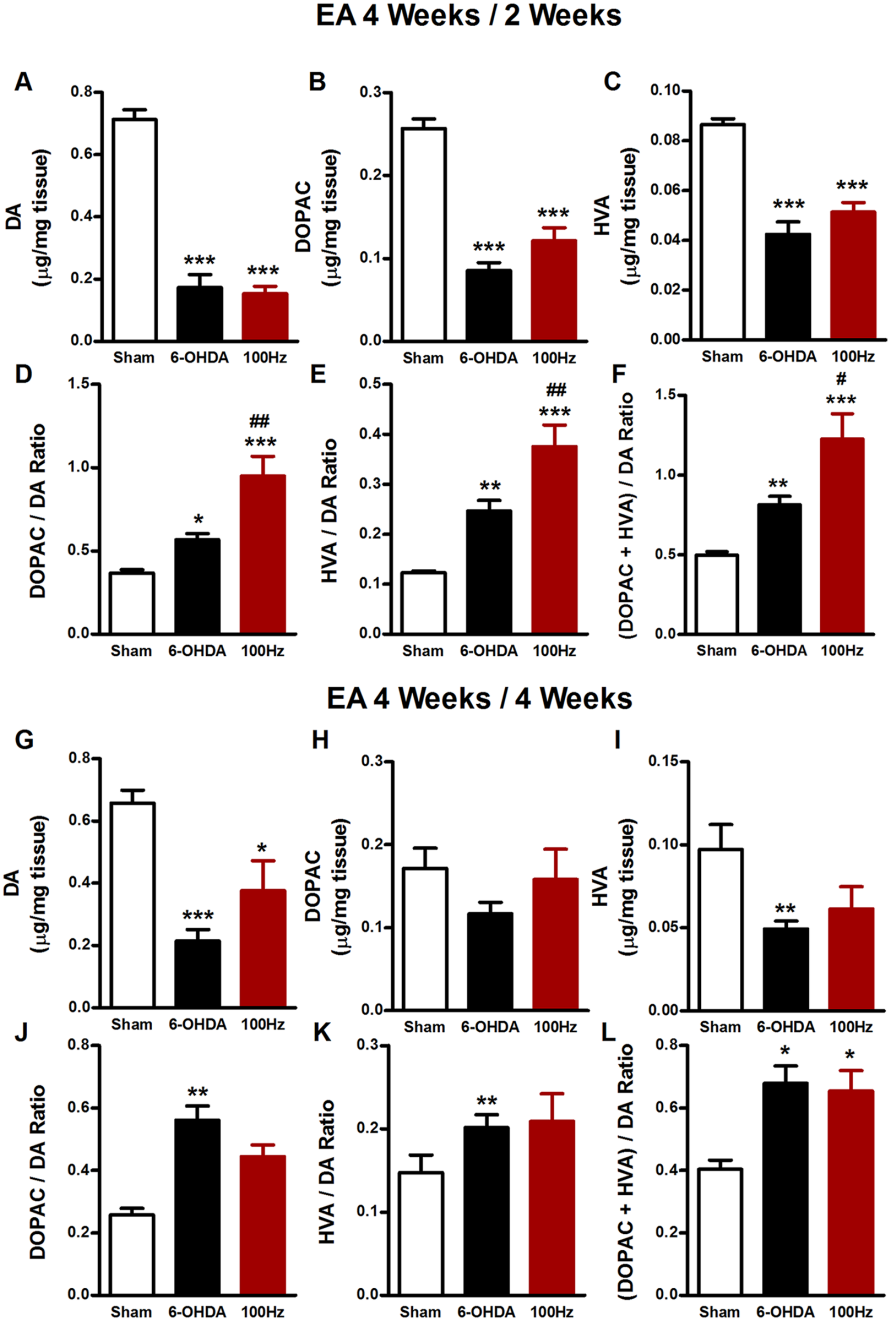
Dopamine and its metabolites in the striatum at the 2^nd^ and 4^th^ week after the termination of EA stimulation in 6-OHDA-lesioned mice. Contents of DA(A), DOPAC(B), HVA(C), and turnover ratios of DOPAC/DA (D), HVA/DA (E), (DOPAC plus HVA)/DA (F) in the striatum were analyzed at the end of 2^nd^ week after the termination EA treatment (EA 4 Weeks / 2 Weeks). DA (G), DOPAC (H), HVA (I), DOPAC/DA (J), HVA/DA (K), (DOPAC plus HVA)/DA (L) were analyzed at the end of 4^th^ week after the termination of four-week EA stimulation (EA 4 Weeks / 4 Weeks). The values were expressed as means ± SEM. n = 7 per group. * *P* <0.05; ** *P* <0.01; *** *P* <0.001 *vs*. Sham group. # *P* < 0.05; ## *P* <0.01 *vs*. 6-OHDA group.

Previous studies suggest that EA exerted its improvement of motor dysfunction during its therapeutic stage through enhancing the dopamine metabolism but not restoring dopamine in PD mice [10,21]. To determine whether this is the case for the sustained effect of EA, we measured changes in the content of striatal DA and its metabolites DOPAC and HVA in the lesioned hemisphere with HPLC 2 and 4 weeks after the termination of EA stimulation. As shown in Fig 5, 6-OHDA induced a marked reduction of the concentration of DA (0.172 ± 0.041 μg/mg tissue of 6-OHDA group *vs*. 0.713 ± 0.030 μg/mg tissue of sham group, *P* < 0.001, Fig 5A) and its metabolites DOPAC (0.085 ± 0.009 μg/mg tissue of 6-OHDA group *vs*. 0.257 ± 0.011 μg/mg tissue of sham group, *P* < 0.001, Fig 5B) and HVA (0.042 ± 0.002 μg/mg tissue of 6-OHDA group *vs*. 0.086 ± 0.005 μg/mg tissue of sham group, *P*<0.001, Fig 5C). 100 Hz EA had no influence on levels of DA, DOPAC and HVA at 2 weeks after the completion of 4-week EA treatment. However, EA at 100 Hz significantly increased dopamine turnover ratios as detected by the DOPAC/DA ratio (0.950 ± 0.118 of EA group *vs*. 0.567 ± 0.036 of sham group, *P* < 0.01, Fig 5D), the HVA/DA ratio (0.375 ± 0.043 of EA group *vs*. 0.247 ± 0.021 of sham group, *P* < 0.01, Fig 5E), and the (DOPAC + HVA)/DA ratio (1.227 ± 0.158 of EA group *vs*. 0.814 ± 0.105 of sham group, *P* < 0.05, Fig 5F). Similar to the results observed at 2 weeks after the cessation of EA stimulation, EA had no effect on the reduced levels of DA, DOPAC and HVA in the striatum at 4 weeks after the cessation of EA (Fig 5G–5I). However, at this time point, EA did not alter dopamine turnover ratios as measured by ratios of DOPAC/DA (Fig 5J), HVA/DA (Fig 5K), and (DOPAC + HVA)/DA (Fig 5L). These results indicate that EA increases the dopamine turnover ratio at the early stage of EA absence (2 weeks), which may be related at least in part to the therapeutic effect of EA seen at this stage.

## Discussion

The morphometric and functional observations from the present study demonstrate that chronic EA treatment (30 min EA session, once a day, 6 days a week for 2 or 4 weeks) at both ST36 and SP6 attenuated the motor deficiency and conferred neuroprotection in 6-OHDA-lesioned mice. More importantly, the beneficial effect of EA outlived the duration of EA application as demonstrated by the finding that EA continued to improve motor function two and four weeks after the termination of EA. Similarly, EA persisted to enhance dopamine availability two weeks after the completion of EA application. These results provide evidence for the existence of the sustained therapeutic effect of EA in a mouse model of PD.

### EA improves motor function in a time-dependent manner

The unilateral 6-OHDA lesion of the nigrostriatal pathway is a common PD model widely used to assess the efficacy of anti-PD therapy [22–25]. In this study, we used this model to compare the effect of EA at different time points after EA application. We found that chronic EA treatment for two or four weeks certainly produced beneficial effects in both motor function and dopamine availability. This solidifies our previous studies that chronic EA treatment possesses the ability to alleviate motor dysfunction in PD animals [9,10,14,16]. We believe that EA application in this study caused little or no stress since, during the entire course of EA stimulation, mice showed no stress-like behaviors such as vocalization and hindlimbs flinches.

In addition to above observations, an important finding in this study is that chronic EA treatment produced a long-lasting effect after the completion of EA stimulation. In all three behavioral tests, EA was able to continue to recover motor deficits at 2 weeks after EA the completion of EA stimulation. At 4 weeks after the termination of EA stimulation, behavioral tests still largely showed a significant effect of EA. These results establish the temporal property of the EA effect in improving motor responses to 6-OHDA lesions. Central among this property is that EA produces a long-lasting effect following the termination of EA stimulation.

### Effects of EA on DA metabolism ratio

Many studies have suggested that EA stimulation exerts the motor improvement without restoration of the striatal DA content in PD models [10,26]. Similarly, our results showed no effect of EA on the DA content. While EA has a minimal impact on DA itself, EA may affect DA metabolites to achieve its action. Indeed, Kim *et al*. recently reported that EA, while it did not restore striatal DA levels, increased dopamine turnover ratio, which seems to contribute to the motor effect of EA [21]. In the present study, we analyzed the two DA metabolites: DOPAC which reflects intraneuronal metabolism of DA, and HVA which is a product of intra- and extra-neuronal metabolism of released DA [27]. We calculated DOPAC/DA, HVA/DA, and (DOPAC+HVA)/DA ratios as those ratios closely reflect the metabolism and release of DA [21,27]. We found that EA enhanced these ratios at 2 weeks after the termination of EA stimulation. Therefore, it seems that, at the early stage of EA withdrawal, EA persisted its ability to increase DA release. Released DA might bind to postsynaptic DA receptors and improve motor function after the termination of EA treatment [21]. However, it is noted that EA had some motor improvement, but did not significantly affect the dopamine turnover ratios at a later time point (4 weeks) after the termination of EA. This indicates that some other molecular mechanism(s) may mediate the effect of EA at 4 weeks, which may involve glutamate and GABA as we have reported previously [26]. Future studies will be needed to illustrate the exact mechanisms underlying the sustained EA effect.

Additionally, Meissner *et al*. reported that DA metabolite concentrations may be linked to dopaminergic neuronal firing [28]. This is because DBS simultaneously increased striatal DA metabolite concentrations and firing frequency of nigral DA neurons, but failed to alter the DA content [28]. It is well known that DBS exerts its powerful therapeutic effect on PD patients through modulating the basal ganglia circuit. We have previously reported that EA improves motor symptoms in PD rats through restoring homeostasis of the basal ganglia circuit, especially the direct pathway [29]. Future studies will investigate whether and how the basal ganglia undergo long-lasting plastic changes at different periods of time after the termination of EA stimulation.

## Conclusion

In conclusion, our longitudinal analysis of the effect of EA in a 6-OHDA mouse model demonstrates that EA exhibited the sustained therapeutic effect on the PD-related motor syndrome after the cessation of EA treatment. An increase in the dopamine turnover ratio in the striatum was seen at 2 weeks after the termination of EA stimulation, which corresponds well with the motor improvement seen at the same time.
